# Risk of lower extremity amputations in patients with type 2 diabetes using sodium-glucose co-transporter 2 inhibitors

**DOI:** 10.1007/s00592-021-01805-8

**Published:** 2021-10-05

**Authors:** Spela Zerovnik, Mitja Kos, Igor Locatelli

**Affiliations:** Department of Social Pharmacy, University of Ljubljana, Faculty of Pharmacy, Askerceva cesta 7, 1000 Ljubljana, Slovenia

**Keywords:** Amputation, Type 2 diabetes, Sodium-glucose co-transporter 2 inhibitor, Dipeptidyl peptidase-4 inhibitor

## Abstract

**Aims:**

To compare the influence of sodium-glucose co-transporter 2 inhibitors (SGLT2i) and dipeptidyl peptidase-4 inhibitors (DPP-4i) on the risk of lower extremity amputations in patients with type 2 diabetes in Slovenia.

**Methods:**

This retrospective cohort study included patients aged 40 years or more who were administered a newly introduced SGLT2i or DPP-4i between June 2014 and June 2018. Patients treated with insulin at baseline and patients with a history of amputation were excluded. Patients were matched in a 1:1 ratio using propensity score matching. Survival analysis was performed; hazard ratio (HR) and ratios of cumulative hazards at 1, 2, 3, and 4 years were estimated. On-treatment and intention-to-treat approaches were used.

**Results:**

The study cohort (mean age: 64 years) consisted of 2,939 new users of SGLT2i (empagliflozin, 59%; dapagliflozin, 41%) matched to 2,939 new users of DPP-4i. In the on-treatment analysis (median follow-up of 2 years), the incidence of amputations was higher in SGLT2i than in DPP-4i users (4.2 vs. 2.7 per 1,000 patient years), resulting in a HR of 1.58 (95% CI 0.85–2.92; *p* = 0.145). An intention-to-treat analysis yielded to similar HR of 1.86 (95% CI: 1.10–3.14; *p* = 0.020). There was no difference in amputation rates in the first two years, but SGLT2i users had a 2.81-fold higher (95% CI: 1.63–4.84; *p* = 0.007) cumulative hazard of amputation at 4 years than did DPP-4i users.

**Conclusions:**

Compared with DPP-4i use, SGLT2i use did not result in a statistically significant higher overall risk of lower extremity amputations. However, the results suggest that SGLT2i may increase the risk of amputation with long-term use.

**Supplementary Information:**

The online version contains supplementary material available at 10.1007/s00592-021-01805-8.

## Introduction

Randomised controlled trials indicate that sodium-glucose co-transporter 2 inhibitors (SGLT2i) may reduce the risk of cardiovascular and renal events in patients with type 2 diabetes [1–6]. However, results from the Canagliflozin Cardiovascular Assessment Study (CANVAS) program [1] have raised safety concerns regarding a potentially increased risk of lower extremity amputations (LEA) in patients using canagliflozin, compared with patients using placebo (hazard ratio [HR] estimate 1.97; 95% confidence interval [CI] 1.41–2.75). Although randomised controlled trials of other SGLT2i have not reported similar associations [3–6], it remains unknown whether this is a class effect. In response to results from the CANVAS Program, the European Medicines Agency issued a safety communication in 2017 on the potential increased risk of LEA in patients taking any SGLT2i [7]. Consequently, a warning about the potential increased risk of LEA was included in the prescribing information for all SGLT2i.

Other observational studies have shown an increased risk of LEA in patients using the SGLT2i empagliflozin or dapagliflozin [8, 9]. For example, a cohort study by Ueda et al. [9] showed a more than twofold increased risk of LEA (median follow-up of 0.7 years) in patients using empagliflozin or dapagliflozin, compared with patients using glucagon-like peptide-1 receptor agonists (GLP-1RA). However, studies using dipeptidyl peptidase-4 inhibitors (DPP-4i) as a comparator showed conflicting findings [8, 10–13]. These conflicting results could be due to differences in study design and exclusion criteria, such as the exclusion of patients at higher risk of amputation (e.g. those treated with insulin at baseline or with a history of amputation). Also, although these studies included a large number of real-world patients with type 2 diabetes, the relatively short follow-up of less than 1 year may have precluded estimation of potential long-term effects of SGLT2i on the incidence of LEA. In addition, canagliflozin was by far the most commonly prescribed SGLT2i in these studies, so additional studies are needed to assess the risk of LEA in patients using other SGLT2i. Of the SGLT2i drug class, only empagliflozin and dapagliflozin were available in Slovenia during the data analysis period of this study.

The aim of the study was to compare the risk of LEA in patients with type 2 diabetes who were treated with SGLT2i (empagliflozin or dapagliflozin) to those treated with DPP-4i.

## Methods

We conducted a retrospective cohort study using an active comparator, new-user design [14]. We used DPP-4i as the reference category, as this drug class is used similarly to SGLT2i as a second- or third-line therapy in patients with type 2 diabetes.

### Data sources

We used three National Institute of Public Health (NIJZ) databases from Slovenia (Outpatient Prescription Medicines Database, National Hospital Health Care Statistics Database, and Causes of Death Registry) for the period 2009 to 2019. The databases, respectively, contain information on outpatient prescription claims, hospitalisation claims, and patient death for the entire Slovenian population (a detailed description of the databases is provided elsewhere [15]). The study protocol was registered at the European Network of Centres for Pharmacoepidemiology and Pharmacovigilance (registration number: EUPAS32558). The National Medical Ethics Committee approved the protocol (registration number: 0120-264/2019/5).

### Cohort selection

The database population consisted of all patients who filled at least two prescriptions for any antidiabetic medicine within 1 year prior to 30 June 2014. To minimise inclusion of patients with type 1 diabetes, gestational diabetes, or early-onset type 2 diabetes, we limited patient selection to those aged 40 years or older. From this patient population, we selected new users of SGLT2i or DPP-4i between 30 June 2014 and 30 June 2018 (recruitment period). To limit the sample to new users of SGLT2i or DPP-4i, we excluded patients treated with SGLT2i, DPP-4i, or GLP-1RA at any time before the index date. The inclusion period started on 30 June 2014, the date when the first drug from the SGLT2i group became available on the Slovenian market.

**Fig. 1:**
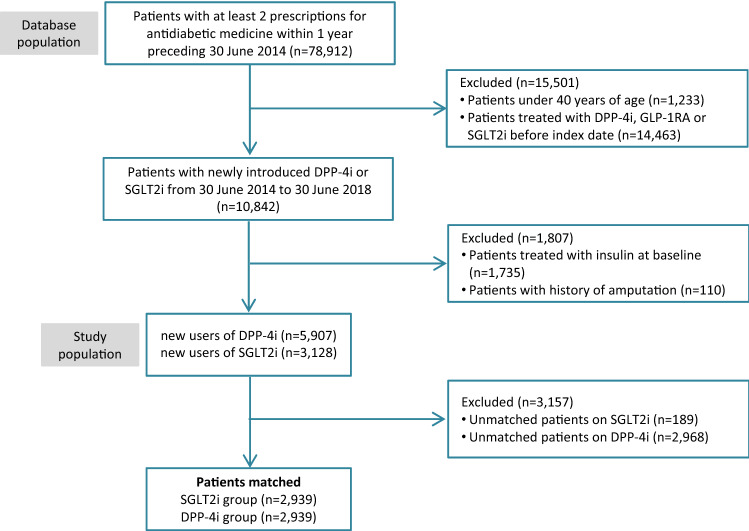
Flowchart of cohort selection. DPP-4i-dipeptidyl peptidase-4 inhibitor; GLP-1RA-glucagon-like peptide-1 receptor agonist; SGLT2i-sodium-glucose co-transporter 2 inhibitor

We defined the date of cohort entry (index date) as the date on which the patient filled the first prescription for SGLT2i or DPP-4i (see supplementary Table S1 for a list of Anatomical Therapeutic Chemical codes defining exposure to either study drug). In Slovenia, insulin is prescribed more frequently in patients starting SGLT2i than in patients starting DPP-4i. Inclusion of patients treated with insulin could result in less comparable treatment groups, as these patients are at higher risk of LEA than patients treated with non-insulin antidiabetic medicines [16]. Thus, we excluded patients treated with insulin on the index date. We also excluded patients with a history of LEA, as these patients are at higher risk for subsequent amputation [17]. Baseline characteristics of the study cohort were evaluated at the index date (see supplementary Table S2 for definitions of the baseline characteristics). Figure 1 presents the flowchart of cohort selection.

### Outcome definition

The outcome under this study was non-traumatic LEA. Therapeutic and diagnostic procedure codes describing minor, ankle-level, and major amputations were used to define the study outcome (see supplementary Table S3). In Slovenia, the Australian Classification of Health Interventions, sixth edition, has been used to code therapeutic and diagnostic procedures since 1 January 2013. Our analysis included only the first LEA event after the index date. The study period ended on 31 December 2019.

### Propensity score matching

The propensity score model comprised 44 variables including patient demographics, such as age (categorical variable with 5-year groupings) and gender; duration of diabetes therapy, which was defined as the time between first prescription of an antidiabetic medicine (after 1 January 2009) and the index date (categorical variable with 2 categories: less than 5 years, 5 years or more); baseline antidiabetic therapy (antidiabetic medicines used in the 135 days before the index date); prior hospitalisations; and concomitant therapy (other medicines used in the 135 days before the index date). Table 1 shows all variables included in the propensity score model.

**Table 1:** Patient characteristics before and after propensity score matching

	Before propensity score matching			After propensity score matching		
Characteristic	SGLT2i (*n* = 3,128)	DPP-4i (*n* = 5,907)	SMD	SGLT2i (*n* = 2,939)	DPP-4i (*n* = 2,939)	SMD
Gender (female)	1,218 (38.9)	2,932 (49.6)	− 0.22	1,200 (40.8)	1,162 (39.5)	0.03
Patient age (mean, SD)	63.7 (8.7)	69.3 (11.2)	− 0.56	64.04 (8.8)	63.99 (8.9)	0.01
*Duration of diabetes therapy*						
Less than 5 years	676 (21.6)	1,398 (23.7)	− 0.05	671 (22.8)	654 (22.3)	0.01
5 years or more	2,452 (78.4)	4,509 (76.3)	0.05	2,268 (77.2)	2,285 (77.7)	− 0.01
*Antidiabetic medicines used in the past 135 days*						
Metformin	2,801 (89.5)	4,646 (78.7)	0.30	2,612 (88.9)	2,594 (88.3)	0.02
Sulphonylureas	2,675 (85.5)	4,676 (79.2)	0.17	2,486 (84.6)	2,480 (84.4)	0.01
Repaglinide	87 (2.8)	224 (3.8)	− 0.06	85 (2.9)	85 (2.9)	< 0.01
*Previous hospitalisation*						
Hospital admission due to CV causes in the past year	180 (5.8)	460 (7.8)	− 0.08	162 (5.5)	161 (5.5)	< 0.01
Hospital admission due to type 2 diabetes in the past year	16 (0.5)	90 (1.5)	− 0.10	16 (0.5)	11 (0.4)	0.02
Hospital admission due to/with cancer in the past five years	101 (3.2)	340 (5.8)	− 0.12	101 (3.4)	90 (3.1)	0.02
*Concomitant therapy (other medicines used in the past 135 days)*						
Medicine for acid-related disorder	804 (25.7)	1,816 (30.7)	− 0.11	763 (26.0)	745 (25.3)	0.01
Anticoagulant	220 (7.0)	688 (11.6)	− 0.16	209 (7.1)	201 (6.8)	0.01
Platelet inhibitor	1,070 (34.2)	2,095 (35.3)	− 0.03	975 (33.2)	983 (33.4)	− 0.01
Cardiac glycoside (metildigoxin)	49 (1.6)	210 (3.6)	− 0.13	48 (1.6)	43 (1.5)	0.01
Antiarrhythmic	26 (0.8)	89 (1.5)	− 0.06	26 (0.9)	32 (1.1)	− 0.02
Vasodilator	99 (3.2)	307 (5.2)	− 0.10	97 (3.3)	96 (3.3)	< 0.01
Loop diuretic	236 (7.5)	850 (14.4)	− 0.22	231 (7.9)	217 (7.4)	0.02
Thiazide and other diuretic	1,313 (42.0)	2,599 (44.0)	− 0.04	1,253 (42.6)	1,239 (42.2)	0.01
MRA	122 (3.9)	294 (5.0)	− 0.05	104 (3.5)	113 (3.8)	− 0.02
Beta blocker	1,202 (38.4)	2,358 (39.9)	− 0.03	1,076 (36.6)	1,084 (36.9)	− 0.01
Calcium channel blocker	1,090 (34.8)	2,108 (35.7)	− 0.02	1,009 (34.3)	1,016 (34.6)	− 0.01
ACE-inhibitor or ARB	2,306 (73.7)	4,247 (71.9)	0.04	2,129 (72.4)	2,130 (72.5)	< 0.01
Statin	1,929 (61.7)	3,383 (57.3)	0.09	1,763 (60.0)	1,789 (60.9)	− 0.02
Other lipid modifying drug	173 (5.5)	272 (4.6)	0.04	167 (5.7)	167 (5.7)	< 0.01
Oral glucocorticoid	24 (0.8)	114 (1.9)	− 0.10	24 (0.8)	22 (0.7)	0.01
Thyroid hormone	179 (5.7)	374 (6.3)	− 0.03	166 (5.6)	165 (5.6)	< 0.01
Antibiotic	478 (15.3)	1,128 (19.1)	− 0.10	473 (16.1)	438 (14.9)	0.03
NSAID	713 (22.8)	1,231 (20.8)	0.05	672 (22.9)	664 (22.6)	0.01
Opioid	269 (8.6)	690 (11.7)	− 0.10	255 (8.7)	256 (8.7)	< 0.01
Antipsychotic	101 (3.2)	322 (5.5)	− 0.11	101 (3.4)	90 (3.1)	0.02
Anxiolytic, hypnotic, or sedative	392 (12.5)	965 (16.3)	− 0.11	366 (12.5)	368 (12.5)	< 0.01
Pregabalin and/or gabapentin	88 (2.8)	196 (3.3)	− 0.03	83 (2.8)	91 (3.1)	− 0.02
TCA	9 (0.3)	23 (0.4)	− 0.02	9 (0.3)	10 (0.3)	− 0.01
Duloxetine and/or venlafaxine	78 (2.5)	171 (2.9)	− 0.02	72 (2.4)	76 (2.6)	− 0.01
SSRI	191 (6.1)	464 (7.9)	− 0.07	189 (6.4)	181 (6.2)	0.01
Medicine for obstructive airway diseases	265 (8.5)	527 (8.9)	− 0.02	237 (8.1)	226 (7.7)	0.01

*ACE* Angiotensin-converting enzyme inhibitor, *ARB* Angiotensin II receptor blocker, *CV* Cardiovascular, *DPP-4i* Dipeptidyl peptidase-4 inhibitor, *IQR* Interquartile range, *MRA* Mineralocorticoid (aldosterone) receptor antagonist, *NSAID* Nonsteroidal anti-inflammatory medicine, *SD* Standard deviation, *SGLT2i* Sodium-glucose co-transporter 2 inhibitor, *SMD* Standardised mean difference, *SSRI* Selective serotonin reuptake inhibitor, *TCA* Tricyclic antidepressant

Propensity scores were estimated using a logistic regression model. Patients were matched on the logit of the propensity score using a calliper width of 0.2 of the pooled standard deviation of the logit of the propensity score [18]. The nearest neighbour matching algorithm without replacement was used to match patients treated with SGLT2i to those treated with DPP-4i in a 1:1 ratio. The balance between covariates in the matched sample was assessed using standardised differences. If the absolute value of the standardised difference was less than 0.10, the respective variable was considered successfully matched [19]. We also performed test of balancing property for each variable.

### Statistical analysis

An on-treatment approach with a 180-day grace period was used for the primary analysis. Patients were followed from the index date until they discontinued treatment (allowing for a 180-day grace period after the end of the days’ supply), or switched to a comparator drug, or were censored on the date of death, or the end of the study period (31 December 2019). We also analysed data using the intention-to-treat (ITT) approach, in which patients were followed until death or the end of the study period, irrespective of any changes in their antidiabetic therapy that occurred after the index date. We also repeated the primary analysis using grace periods of 90 and 365 days to define continuous use of the newly introduced drug. In an additional analysis, we considered the use of insulin after the index date as a time-dependent variable.

We estimated the hazard ratios (HR) for amputations among new users of SGLT2i compared with new users of DPP-4i using Cox proportional hazards regression models with robust variance estimator [20]. The proportional hazards assumption test based on the Schoenfeld residuals was used to test whether the proportional hazards assumption of the Cox models was satisfied.

In addition, we estimated 1-, 2-, 3-, and 4-year cumulative probabilities for amputations separately for SGLT2i and DPP-4i group [21]. The 95% CIs for cumulative probabilities were calculated based on the log minus log (CLOGLOG) transformation of the survival function. The differences in cumulative probabilities between SGLT2i and DPP-4i group at specific time points were tested using Wald test based on a CLOGLOG transformation of the survival function [22]. To numerically represent the magnitude of these differences, we also calculated the ratios of cumulative hazards (RCH) for amputations between SGLT2i and DPP-4i group with the corresponding 95% CIs at specific time points.

*P* values less than 0.05 indicated statistical significance. Data preparation and statistical analysis were performed using IBM SPSS Statistics for Windows, Version 27.0 (Armonk, NY, USA: IBM Corp) and Stata Statistical Software: Release 16 (College Station, TX, USA: StataCorp LLC). The cumulative probability curves (i.e. 1 minus survival curve) for amputations were plotted in R-4.0. software environment (packages Survival and Survminer).

## Results

We identified 10,842 new users of DPP-4i or SGLT2i from 30 June 2014 to 30 June 2018. Of those, we excluded 1,735 (16%) who were treated with insulin. We also excluded 110 patients who had a history of LEA. The final cohort comprised 3,128 new users of SGLT2i and 5,907 new users of DPP-4i. Prior to propensity score matching, we observed some differences in baseline patient characteristics, including age, proportion of female patients, use of metformin and sulphonylureas at baseline, prior hospitalisations, and use of several concomitant medications. However, after applying propensity score matching, the groups were well balanced (absolute values of standardised differences between covariates in the matched sample were less than 0.05) with respect to all baseline characteristics included in the propensity score model. After matching, our study cohort consisted of 2,939 new users of SGLT2i (empagliflozin, 59%; dapagliflozin, 41%) and 2,939 new users of DPP-4i (sitagliptin, 64%; linagliptin, 24%; vildagliptin, 11%; saxagliptin and alogliptin, less than 1%). Table 1 presents baseline characteristics of the study cohort before and after matching.

**Fig. 2:**
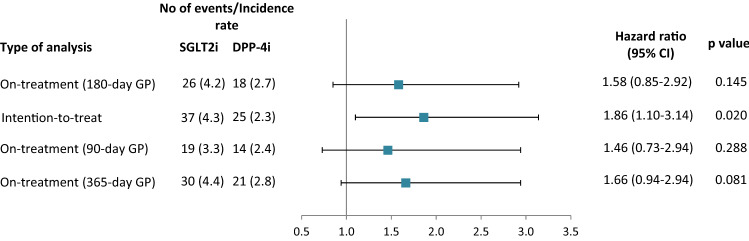
Hazard ratio estimates for the association between the use of SGLT2i compared with the use of DPP-4i and the risk of lower extremity amputations. Incidence rate-number of events per 1,000 patient years; DPP-4i-dipeptidyl peptidase-4 inhibitor; GP-grace period; SGLT2i-sodium-glucose co-transporter 2 inhibitor

We identified 44 LEA events over a median (interquartile range; IQR) follow-up of 2.0 (1.0–3.1) years in the on-treatment analysis. In the ITT analysis, there were 62 LEA events over a median (IQR) follow-up of 3.3 (2.3–4.2) years. More than 80% of amputations were minor (below the ankle). The incidence rate of amputations in the on-treatment analysis was higher among new users of SGLT2i (4.2 per 1,000 patient years), compared to new users of DPP-4i (2.7 per 1,000 patient years), resulting in an HR of 1.58 (95% CI 0.85–2.92; *p* = 0.145). The incidence rate of events in the ITT analysis was similar to that in the on-treatment analysis (4.3 and 2.3 per 1,000 patient years among new users of SGLT2i and DPP-4i, respectively) with a corresponding HR of 1.86 (95% CI 1.10–3.14; *p* = 0.020). The results of the sensitivity analyses using grace periods of 90 and 365 days after the last prescription were similar to those in the primary analysis. HR estimates slightly decreased when the 90-day grace period was used and increased when the 365-day grace period was used. Figure 2 illustrates the results.

The results of the model in which the use of insulin was treated as a time-dependent variable were consistent with the results of the primary analysis (HR = 1.61; 95% CI 0.87–2.96; *p* = 0.127). At the time of the event or at the time of censoring, 12% of patients in the SGLT2i group and 18% of patients in the DPP-4i group were prescribed insulin.

**Fig. 3:**
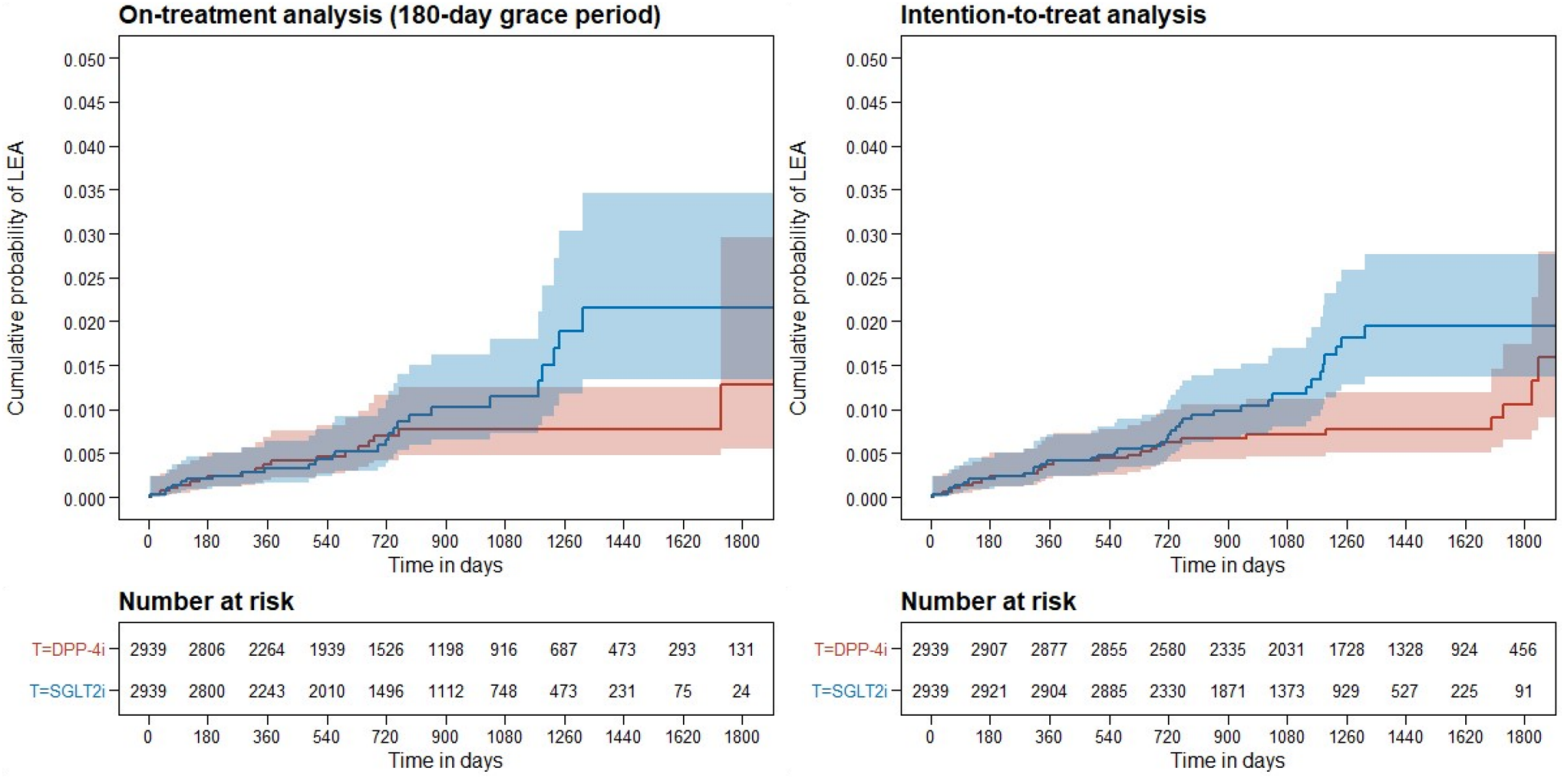
The cumulative probability of lower extremity amputations for on-treatment analysis (180-day grace period) and intention-to-treat analysis. Blue curve represents SGLT2i group, and the red curve represents DPP-4i group; DPP-4i-dipeptidyl peptidase-4 inhibitor; LEA-lower extremity amputation; SGLT2i-sodium-glucose co-transporter 2 inhibitor; T-treatment group

The proportional hazards assumptions of Cox models were marginally violated (*p* values around 0.05) for both the on-treatment (180-day grace period) and ITT analyses; therefore, we also estimated 1-, 2-, 3-, and 4-year cumulative probabilities of LEAs for each treatment group (Table 2). Cumulative probability curves are plotted in Fig. 3. In both, on-treatment and ITT analyses, there was no difference in LEA rates between groups during the first 2 years. However, at 4 years, there was a statistically significantly higher probability of LEAs in the SGLT2i group compared with the DPP-4i group (Table 2). The ratio of cumulative hazards (95% CI) for LEAs was 2.81 (1.63–4.84) in the on-treatment analysis and 2.53 (1.45–4.44) in the ITT analysis. The results of the sensitivity analyses were similar. Results for the on-treatment analyses using 90- and 365-day grace periods are presented in Supplementary Table S4 and Supplementary Fig. S1.

**Table 2:** Cumulative probabilities and 95% confidence intervals of lower extremity amputations for SGLT2i and DPP-4i at different points in time

		Time point			
	Median (IQR) follow-up (yrs)	1 year	2 years	3 years	4 years
*On-treatment (180-day grace period)*					
SGLT2i	2.01 (1.03–3.00)	0.33% (0.17–0.63%)	0.66% (0.39–1.11%)	1.14% (0.72–1.80%)	2.15% (1.33–3.47%)
DPP-4i	2.05 (1.05–3.32)	0.37% (0.20–0.69%)	0.70% (0.42–1.15%)	0.77% (0.47–1.25%)	0.77% (0.47–1.25%)
RCH (95% CI),* p* value	/	0.89 (0.36–2.20) 0.804	0.94 (0.45–1.95) 0.874	1.48 (0.76–2.91) 0.251	2.81 (1.63–4.84) **0.007**
*Intention-to-treat*					
SGLT2i	2.88 (2.11–3.70)	0.41% (0.23–0.72%)	0.71% (0.46–1.10%)	1.17% (0.81–1.70%)	1.94% (1.36–2.77%)
DPP-4i	3.79 (2.69–4.65)	0.38% (0.21–0.68%)	0.63% (0.40–1.00%)	0.72% (0.46–1.11%)	0.77% (0.50–1.18%)
RCH (95% CI),* p* value	/	1.08 (0.47–2.45) 0.856	1.13 (0.60–2.13) 0.711	1.63 (0.91–2.90) 0.098	2.53 (1.45–4.44) **0.001**

Bold *p* values are statistically significant

*CI* Confidence interval, *DPP-4i* Dipeptidyl peptidase-4 inhibitor, *IQR* Interquartile range, *LEA* Lower extremity amputation, *RCH* Ratio of cumulative hazards, *SGLT2i* Sodium-glucose co-transporter 2 inhibitor

### Discussion

The current study, in which patients with type 2 diabetes were followed for a median of 2 years in the primary analysis, showed that SGLT2i use, compared with DPP-4i use did not result in a statistically significant higher overall risk of LEA. The results of this analysis were borderline significant, possibly because the analysis was underpowered despite the fact that we used a quite long recruitment period. Moreover, the hazards in the Cox models were non-proportional, suggesting that the effect of treatment groups on LEA risk varies over time. In case of non-proportional hazards, the power of the log-rank test for comparing the entire survival curves is low; therefore, it is more appropriate to compare survival curves (or cumulative probability curves) at fixed time points [21]. The comparison of cumulative probability curves revealed no difference in the first 2 years (overlapping curves), but at around 2 years the cumulative probability curves started to diverge. At 4 years, SGLT2i users had a statistically significant 2.8-fold higher cumulative hazard of LEA than DPP-4i users, suggesting that the increased amputation risk may occur with long-term use of SGLT2i.

The results of the ITT analysis and sensitivity analyses (on-treatment analyses using 90- and 365-day grace periods) were similar to those of the primary analyses. In contrast with the on-treatment analysis, the ITT analysis also showed statistically significant differences between groups in the Cox proportional regression model. The results of the Cox model for the ITT analysis may be significant because it included a higher number of events per treatment group than the primary analysis. In the primary analysis, many patients discontinued the study drug or switched to the study comparator during follow-up, resulting in a smaller number of events.

Although patients treated with insulin at baseline were excluded from the analysis, approximately 15% of patients started insulin during follow-up. The proportion of patients who initiated insulin was higher in the DPP-4i group than in the SGLT2i group (18% vs. 12%), which may have reduced the difference in LEA risk between treatment groups, as insulin is typically prescribed to patients with more severe type 2 diabetes and is associated with a fivefold increased risk of LEA [16]. However, we performed a time-dependent Cox regression analysis that accounted for initiation of insulin during follow-up. The HR estimate from this analysis was slightly higher than that in the primary analysis, suggesting that insulin initiation during follow-up only slightly reduced the difference in LEA risk between groups.

Previous observational studies comparing DPP-4i and SGLT2i provide conflicting results [8, 10–13]. The differences in findings might be due to differences in study populations, methodological approach, inclusion/exclusion of patients with history of amputation or use of insulin at baseline, duration of follow-up, statistical analysis used, and type of SGLT2i prescribed. However, the point estimate of HR in our study is similar to that of two US cohort studies [8, 10], which also exclude patients treated with insulin at baseline. In contrast to our study, most patients in these studies were treated with canagliflozin, and the study by Adimadhyam et al. includes patients with a history of amputation, though the proportion of such patients was negligible (approximately 0.1%). Another study using US data [11] and including both patients using insulin at baseline and patients with a history of amputation yielded similar results, with an HR estimate of 1.69 (95% CI 1.20–2.38). In contrast, two other cohort studies with a median follow-up of approximately 1 year [12, 13] report no difference in LEA risk between users of SGLT2i and users of DPP-4i (HR estimates around 0.88). Both studies include patients treated with insulin at baseline (21–27.5% of the study cohort) and exclude patients with a history of amputation. The study by Yu et al. [13] is the only study to evaluate risk of LEA in which most patients (approximately 60%) use empagliflozin or dapagliflozin; in all other studies, canagliflozin is by far the most commonly prescribed SGLT2i [11, 12].

The incidence rates of LEA in our study exceed those in other observational studies and are similar to incidence rates reported for the placebo arm in the CANVAS Program (3.4 per 1,000 patient years) [1]. Although we excluded patients treated with insulin at baseline (16%) and patients with prior amputation (1%), it appears that our study cohort had a higher baseline risk of amputation. Patients in our cohort were on average 10 years older than patients in similar observational studies [8, 10–12] and were more frequently male. Older age and male gender are known risk factors for LEA [16, 17]. Furthermore, OECD data from 2013 [23, 24] show that Slovenia has one of the highest rates of LEA in patients with diabetes among all countries included in the analysis. Although the results of this study indicate that SGLT2i may increase the risk of LEA in patients with type 2 diabetes, the overall risk of amputation was relatively low. Moreover, the beneficial effects of SGLT2i on cardiovascular and renal outcomes also should be considered when prescribing SGLT2i. The beneficial effect of SGLT2i on cardiovascular morbidity and mortality was also shown in another study that evaluated cardiovascular outcomes of Slovenian patients with type 2 diabetes [15].

Our study has several strengths. It is the first to evaluate LEA risk over a longer period of time (our median follow-up of 2 years is at least 1 year longer than in other studies). This is particularly important as the differences between groups in our study manifested after 2 or more years of treatment. Ours is the only study to suggest that the effect of SGLT2i on LEA risk may be delayed and associated with duration of treatment. The mechanism behind this potentially increased risk of LEA is unclear and should be further explored. Some authors suggest that SGLT2i may increase risk of amputation due to a diuretic effect leading to volume depletion and decreased perfusion of the lower extremities [25, 26]. Our study also offers novel insights on the risk of LEA in users of empagliflozin and dapagliflozin, the most commonly prescribed SGLT2i in the EU market [27, 28]. Although randomised controlled trials of these two agents show no evidence of increased risk of LEA, our study suggests a possible class effect. This finding complements those of the cohort study by Ueda et al. [9], in which the use of empagliflozin or dapagliflozin was associated with a more than twofold increased risk of LEA, compared with the use of GLP-1RA. We used the DPP-4i group as the study comparator because this drug class is used in patients with similar stages of type 2 diabetes. Finally, our results were consistent across all sensitivity analyses.

However, the results of this study must be interpreted in the context of some limitations. First, data on the time of diabetes diagnosis were not included in the databases used in this study and thus omitted from the propensity score model. However, time between first dispensed prescription of an antidiabetic medicine and index date was used to proxy for diabetes therapy duration. Because data prior to 2009 were not available, we could not estimate the exact duration of diabetes therapy, but could only distinguish between patients who had been treated with antidiabetic medicines for more or less than five years. Second, data on other comorbidities (concomitant diagnoses) were not available for all patients included in the cohort, but only for patients admitted to the hospital at least once in the period from 1 January 2009 to 31 December 2019. Therefore, data on outpatient prescriptions were used to proxy for comorbidities. However, because some comorbidities (i.e. cardiovascular disease, microvascular complications of type 2 diabetes) are associated with a higher risk of amputation, we also included data on hospitalisations due to these comorbidities in the year prior to index date in the propensity score model. Third, we were unable to adjust for some potential confounders, such as HbA1c levels or body mass index, because these data were not included in the databases. Fourth, because the number of events was too small, we could not stratify our analysis by the type of SGLT2i used, so we could not determine whether the increased risk of LEA was associated with both empagliflozin and dapagliflozin. Fifth, the validity of therapeutic and diagnostic procedure codes for amputations in hospitalisation claims data was not examined, which may lead to outcome misclassification, though it is unlikely that outcome misclassification differed between treatment groups.

## Conclusion

This study showed that the use of the SGLT2i drugs empagliflozin and dapagliflozin did not result in a statistically significant higher overall risk of LEA in patients with type 2 diabetes compared with the use of DPP-4i. However, the results suggest that SGLT2i may increase the risk of amputation with long-term use. Further studies with long follow-ups including large number of patients are needed to confirm this finding and to investigate whether this applies to all medicines in the SGTL2i drug class.

## Supplementary Information

Below is the link to the electronic supplementary material.

Supplementary file1 (DOCX 166 KB)

## Data Availability

Data were obtained from a third party (National Institute of Public Health – Slovenia, www.nijz.si/en) and are not publicly available.
