# Supplementary material for: Risk of lower extremity amputations in patients with type 2 diabetes using sodium-glucose co-transporter 2 inhibitors
Source: Acta Diabetol. 2021 Oct 5;59(2):233–41. doi: 10.1007/s00592-021-01805-8 (PMC8841312; doi:10.1007/s00592-021-01805-8)
Supplement: Supplementary file 1 — Supplementary file1 (DOCX 166 KB) [file 592_2021_1805_MOESM1_ESM.docx]

**Supplementary material**

## Risk of lower extremity amputations in patients with type 2 diabetes using sodium-glucose co-transporter 2 inhibitors

Zerovnik Spela, Kos Mitja, Locatelli Igor

**Table S1** ATC codes for exposure definition

**Table S2** Definitions of baseline patient characteristics

**Table S3** ACHI codes used to define outcomes

**Table S4** Probabilities and 95% confidence intervals of lower extremity amputations for SGLT2i and DPP-4i at different points in time (for on-treatment analyses with 90- and 365-day grace periods)

**Fig. S1** The cumulative probability of lower extremity amputation for on-treatment analyses with 90- and 365-day grace periods**Table S1** ATC codes for exposure definition

| **Exposure** | **ATC codes** |
| --- | --- |
| Dipeptidyl peptidase-4 inhibitors | A10BH01, A10BH02, A10BH03, A10BH04, A10BH05, A10BD07, A10BD08, A10BD09, A10BD10, A10BD11, A10BD13 |
| Sodium-glucose co-transporter 2 inhibitors | A10BK01, A10BK02, A10BK03, A10BK04, A10BD15, A10BD20 |

ATC – Anatomical Therapeutic Chemical

**Table S2** Definitions of baseline patient characteristics

| **Characteristic** | **Definition** |
| --- | --- |
| **Duration of diabetes therapy** | The time between first prescription of an antidiabetic medicine (after 1 January 2009) and the index date. |
| **Antidiabetic medicines used in the past 135 days** | At least one prescription for an antidiabetic medicine within the 135 days preceding index date |
| *Metformin* | ATC codes: A10BA02, A10BD02, A10BD03, A10BD05, A10BD07, A10BD08, A10BD10, A10BD11, A10BD13, A10BD14, A10BD15, A10BD16, A10BD18, A10BD20, A10BD22, A10BD23, A10BD25 |
| *Sulphonylureas* | ATC codes: A10BB, A10BD02, A10BD04, A10BD06 |
| *Repaglinide* | ATC codes: A10BX02, A10BD14 |
| **Insulin therapy at baseline** | At least one prescription for insulin (ATC code: A10A) in the year preceding index date |
| **Concomitant therapy** | At least one prescription for individual ATC code within the 135 days preceding index date |
| *Medicines for acid-related disorder* | ATC codes: A02 |
| *Anticoagulant* | ATC codes: B01AA, B01AE07, B01AF, B01AX05, B01AB04, B01AB05, B01AB06 |
| *Platelet inhibitor* | ATC codes: B01AC |
| *Cardiac glycoside (metildigoxin)* | ATC codes: C01A |
| *Antiarrhythmic* | ATC codes: C01B |
| *Vasodilator* | ATC codes: C01D |
| *Loop diuretic* | ATC codes: C03C, C03EB |
| *Thiazide and other diuretic* | ATC codes: C03A, C03B, C03EA, C07B, C07C, C07D, C08GA, C09BA, C09BX01, C09BX03, C09DA, C09DX01, C09DX03, C09DX06, C09DX07, C10BX13 |
| *MRA* | ATC codes: C03D, C03E |
| *Beta blocker* | ATC codes: C07, C09BX02, C09BX04, C09DX05 |
| *Calcium channel blocker* | ATC codes: C08C, C08D, C08G, C07FB, C09BB, C09BX01, C09BX03, C09BX04, C09DB, C09DX01, C09DX03, C09DX06, C09DX07, C10BX03, C10BX07, C10BX09, C10BX11, C10BX14 |
| *ACE-inhibitor or ARB* | ATC codes: C09A, C09B, C09C, C09D, C10BX04, C10BX06, C10BX07, C10BX10, C10BX11, C10BX12, C10BX13, C10BX14, C10BX15, C10BX16, C10BX17 |
| *Statin* | ATC codes: C10AA, C10BA, C10BX |
| *Other lipid modifying drug* | ATC codes: C10AB, C10AC, C10AD, C10AX, C10BA |
| *Oral glucocorticoid* | ATC codes: H02AB |
| *Thyroid hormones* | ATC codes: H03AA01, H03AA02, H03AA03 |
| *Antibiotic* | ATC codes: J01 |
| *NSAID* | ATC codes: M01A |
| *Opioid* | ATC codes: N02A |
| *Antipsychotic* | ATC codes: N05A |
| *Anxiolytic, hypnotic, or sedative* | ATC codes: N05B, N05C |
| *Pregabalin and/or gabapentin* | ATC codes: N03AX12, N03AX16 |
| *TCA (amitriptyline)* | ATC codes: N06AA09 |
| *Duloxetine and/or venlafaxine* | ATC codes: N06AX16, N06AX21 |
| *SSRI* | ATC codes: N06AB |
| *Medicine for obstructive airway diseases* | ATC codes: R03 |
| **Previous hospitalisations** | At least one hospital admission due to specific cause (as a main diagnosis) in the year preceding index date |
| *Hospital admission due to cardiovascular causes in the past year* | ICD-10-AM codes: I00-I99 |
| *Hospital admission due to type 2 diabetes in the past year* | ICD-10-AM codes: E11 |
| *Hospital admission with cancer as the main or concomitant diagnosis in the past five years* | ICD-10-AM codes: C00-C97 |
| *History of amputation* | ACHI codes: 4433800, 4435800, 4436400, 4436401, 4436100, 4436101, 4436700, 4436701, 4436702, 4437000, 4437300 |

ACE – angiotensin-converting enzyme inhibitor; ACHI – Australian Classification of Health Interventions; ARB – angiotensin II receptor blocker; ATC – Anatomical Therapeutic Chemical; ICD – International Classification of Diseases, 10th Revision, Australian Modification, Sixth Edition; MRA – mineralocorticoid (aldosterone) receptor antagonist; NSAID – nonsteroidal anti-inflammatory medicine; SSRI – selective serotonin reuptake inhibitor; TCA – tricyclic antidepressant

**Table S3** ACHI codes used to define outcomes

| **Outcome** | **ACHI codes** |
| --- | --- |
| Amputation | 4433800, 4435800, 4436400, 4436401, 4436100, 4436101, 4436700, 4436701, 4436702, 4437000, 4437300 |
| Minor amputation | 4433800, 4435800, 4436400, 4436401 |
| Major amputation | 4436700, 4436701, 4436702, 4437000, 4437300 |
| Ankle-level amputation | 4436100, 4436101 |
| ACHI – Australian Classification of Health Interventions | |

**Table S4** Probabilities and 95% confidence intervals of lower extremity amputations for SGLT2i and DPP-4i group at different points in time (for on-treatment analyses with 90- and 365-day grace periods)

|  | | | **Time point** | | | |  |
| --- | --- | --- | --- | --- | --- | --- | --- |
|  | | **Median (IQR) follow-up (yrs)** | **1 year** | | **2 years** | **3 years** | **4 years** |
| **On-treatment (90-day grace period)** | | | | | | | |
| SGLT2i | | 1.75 (0.66-2.74) | 0.35% (0.18-0.68%) | | 0.68% (0.39-1.17%) | 1.00% (0.59-1.70%) | 1.57% (0.85-2.89%) |
| DPP-4i | | 1.70 (0.73-2.88) | 0.36% (0.19-0.70%) | | 0.56% (0.31-1.00%) | 0.64% (0.36-1.13%) | 0.64% (0.36-1.13%) |
| RCH (95% CI), p value | | / | 0.97 (0.38-2.47)  0.953 | | 1.22 (0.54-2.72)  0.636 | 1.57 (0.72-3.42)  0.262 | 2.46 (1.06-5.72)  **0.036** |
| **On-treatment (365-day grace period)** | | | | | | | |
| SGLT2i | | 2.17 (1.52-3.08) | 0.39% (0.22-0.70%) | | 0.68% (0.42-1.11%) | 1.19% (0.78-1.81%) | 2.25% (1.45-3.50%) |
| DPP-4i | | 2.30 (1.46-3.55) | 0.39% (0.22-0.71%) | | 0.68% (0.42-1.10%) | 0.74% (0.46-1.19%) | 0.74% (0.46-1.19%) |
| RCH (95% CI), p value | | / | 1.00 (0.44-2.28)  1.0 | | 1.00 (0.50-1.99)  1.0 | 1.61 (0.85-3.05)  0.143 | 3.06 (1.59-5.89)  **0.001** |

bold p values are statistically significant; CI – confidence interval; DPP-4i – dipeptidyl peptidase-4 inhibitor; IQR – interquartile range; LEA – lower extremity amputation; RCH – ratio of cumulative hazards; SGLT2i – sodium-glucose co-transporter 2 inhibitor


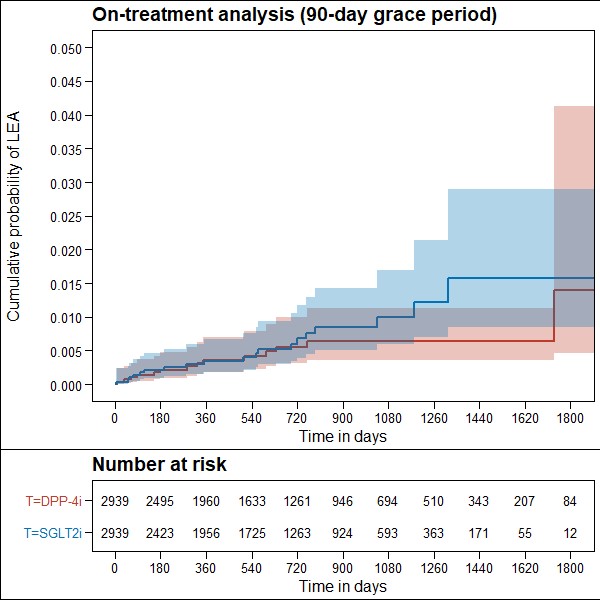

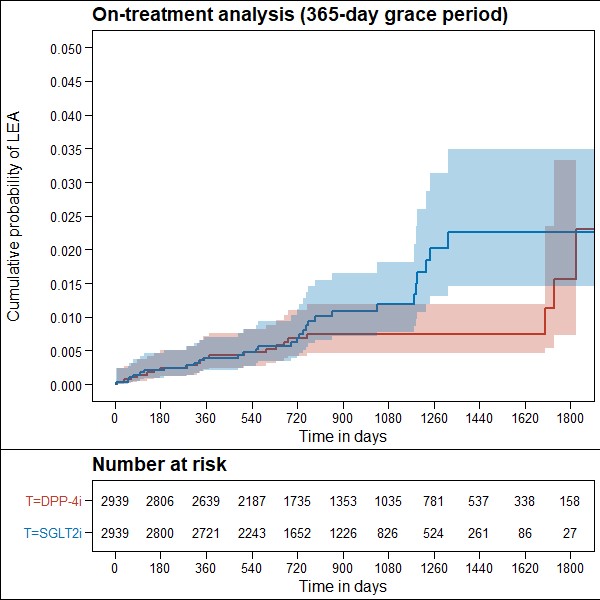


**Fig. S1** The cumulative probability of lower extremity amputation for on-treatment analyses with 90- and 365-day grace periods

Blue curve represents SGLT2i group, and the red curve represents DPP-4i group; DPP-4i – dipeptidyl peptidase-4 inhibitor; LEA – lower extremity amputation; SGLT2i – sodium-glucose co-transporter 2 inhibitor; T – treatment group
